# Iatrogenic Aortocoronary Arteriovenous Fistula following Coronary Artery Bypass Surgery: A Case Report and Complete Review of the Literature

**DOI:** 10.1155/2012/652086

**Published:** 2012-10-31

**Authors:** Jonathan D. Gardner, William R. Maddox, Joe B. Calkins

**Affiliations:** ^1^Internal Medicine Department, Georgia Health Sciences University, Augusta, GA 30912, USA; ^2^Cardiology Department, Georgia Health Sciences University, Augusta, GA 30912, USA; ^3^Cardiology Department, Charlie Norwood VA Medical Center, Augusta, GA 30904, USA

## Abstract

The case of a patient who presented with angina following a coronary artery bypass (CABG) operation during which the left internal mammary artery was inadvertently anastomosed to a cardiac vein is presented. The literature concerning previously reported cases of aortocoronary arteriovenous fistulas (ACAVF) due to inadvertent grafting of a coronary vein is reviewed and the significance of this complication is discussed. ACAVF due to inadvertent grafting of a coronary vein is a rare complication of CABG and may be a more common cause of graft failure than has previously been recognized. Distortion of cardiac anatomy, the presence of epicardial fat, and an intramyocardial course of the artery intended for grafting are predisposing factors. Some patients present with angina pectoris and heart failure whereas others have no symptoms. The diagnostic test of choice is coronary angiography. Cardiac MRI and CT have a limited role due to the smaller size and the more clearly defined course of these fistulas. Asymptomatic patients are simply observed since spontaneous closure of these fistulas is reported. Symptomatic patients can be treated with combined medical management and percutaneous methods.

## 1. Introduction

Iatrogenic aortocoronary arteriovenous fistula (ACAVF) resulting from placement of an arterial graft to a cardiac vein is a rare complication of CABG. We present a case involving grafting of the left internal mammary artery (LIMA) to a left coronary vein and a review of the literature.

## 2. Patient Presentation

The patient is a 69-year-old male with hypertension, hyperlipidemia, and type 2 diabetes mellitus who presented with exertional chest pain, dyspnea, decreased functional capacity and occasional palpitations. A myocardial perfusion study one month earlier had demonstrated inferolateral ischemia and preserved left ventricular systolic function (EF 69%).

**Figure 1:**
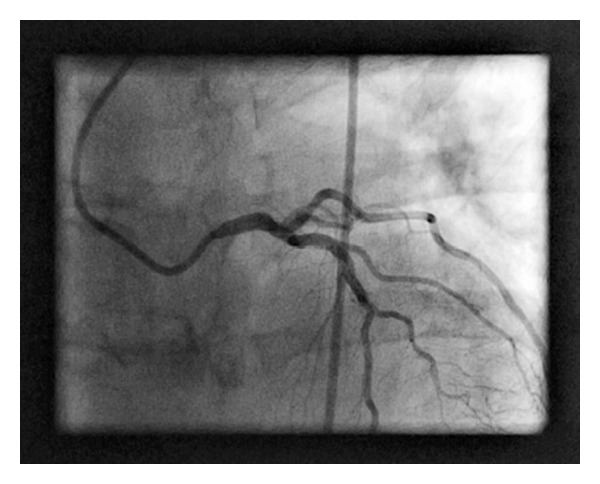
Left anterior oblique view of the left coronary artery. A JL4 catheter is seen engaging the left coronary artery. The ostial left main is tapered and has a 70% stenosis.

Subsequent left heart catheterization (LHC) showed 70% ostial left main stenosis (Figure 1), 60% left anterior descending artery (LAD) stenosis, and complete occlusion of the mid circumflex artery with filling via right to left collaterals. The right coronary artery (RCA) had 70% stenosis in its midportion and left ventricular systolic function was normal. He underwent CABG with the following grafts: LIMA to the LAD, SVG to OM, and SVG to the PDA. Postoperative course was uncomplicated. 

**Figure 2:**
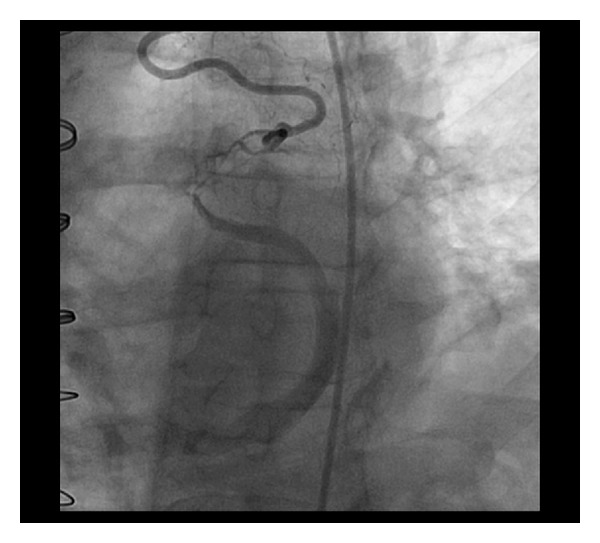
This is an angiogram of the left internal mammary artery as it anastomoses to a cardiac vein. Contrast fills the coronary sinus as it traverses the posterior atrioventricular groove.

Three months later, the patient presented with exertional chest pain similar to his pain prior to surgery. Repeated LHC showed no change in the native coronary arteries and patent SVG to OM and SVG to PDA with good flow. Angiography of the LIMA demonstrated that it was anastomosed to a cardiac vein with resultant flow into the coronary sinus (Figure 2).

**Figure 3:**
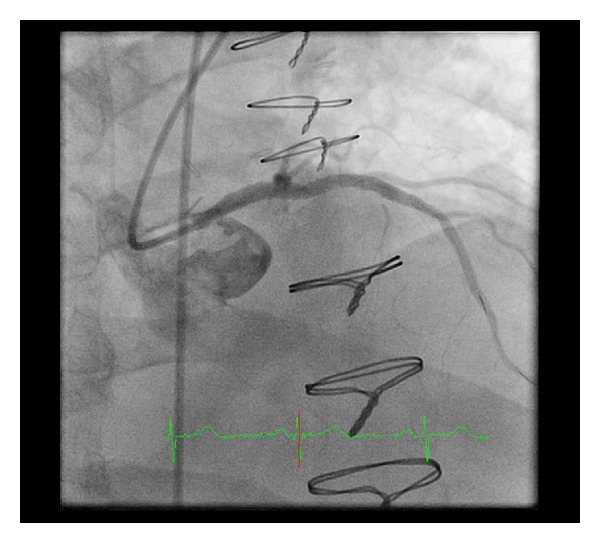
Right anterior oblique view of the left coronary artery following left main and LAD intervention. The left main is no longer tapered and the contrast effluxes out of the left main into the left coronary cusp of the aortic valve.

**Figure 4:**
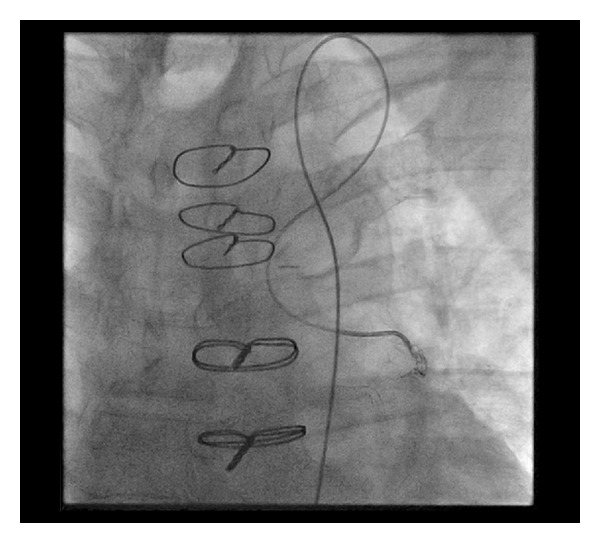
Angiogram of the deployment of the coils in the distal left internal mammary artery. The catheter is accessing the LIMA via the left subclavian artery.

Percutaneous coronary intervention (PCI) was performed with placement of three drug eluting stents in the LM and ostial/proximal LAD. There was no residual stenosis (Figure 3). Subsequently, eight 3 mm stainless steel coils were deployed in the distal portion of the LIMA just proximal to the anastomosis with the cardiac vein with resultant occlusion of the LIMA (Figure 4). 

Six months after the intervention, the patient had a repeated myocardial perfusion study. No ischemia was demonstrated and LV systolic function was normal. He has remained asymptomatic.

## 3. Discussion

Iatrogenic ACAVF resulting from inadvertent grafting to a coronary vein is a rare complication of CABG. Only 36 cases have been reported (Table 1). Deligonul et al. [24] reported two cases of ACAVF, which closed spontaneously, suggesting that this complication may be more frequent than previously thought. Symptomatic patients experiencing spontaneous closure of the ACAVF would be found to have an occluded graft and an unbypassed artery with coronary angiography. It may be unrecognized in other patients with this complication since they may remain asymptomatic or significantly less symptomatic following bypass surgery and would have no indication for coronary angiography.

This complication can result in significant morbidity by several mechanisms. Postoperative angina can occur either as a result of either residual ischemia due to an unbypassed artery or a coronary steal phenomenon. A state of high output failure can result if there is a significant degree of left-to-right shunting over an extended period of time. If left untreated, shunting can cause other complications, such as bacterial endocarditis or fistula rupture [15, 17, 19, 28, 29]. Although significant morbidity may arise, some patients remain asymptomatic and spontaneous closure of the fistula can occur [24].

As this is a rare complication, predisposing factors are difficult to identify. It is reasonable to assume that anatomical distortion of the myocardium may increase the risk of iatrogenic anastomosis of a graft to a cardiac vein. The presence of scarring and fibrosis following pericardial disease, myocardial infarction (MI), or previous CABG as well as the presence of epicardial fat can make identification of the artery more difficult [15, 19, 20, 29]. Thirty-five percent of patients had a previous MI and 18% had undergone CABG. The majority of cases involved the LAD (22 patients, 61%) and its diagonal branch (4 patients, 11%), which can be deeply embedded in epicardial fat or myocardium. 

The majority of patients presented with angina (23 patients, 63.9%) or dyspnea (8 patients, 22.2%) (Table 1). Three presented with heart failure symptoms, two with arrhythmias (VT and SVT), one with syncope, and one with diminished exercise capacity. Four were asymptomatic. No symptoms were reported for another patient who was found to have anterior ischemia on an ECG. The time of onset of symptoms following CABG was variable, ranging from 10 days [8] to 10 years [17]. No obvious trend was present concerning the onset of symptoms following CABG. Symptoms occurred in 14 patients within the first three months postoperatively and between one and 10 years postoperatively in 16 patients. 

The most common physical finding was a continuous or systolic murmur, followed by signs of heart failure (Table 1). However, the majority of reports did not mention whether or not a murmur was present.

In all of the reported cases, the standard diagnostic tool was LHC since it allows direct visualization of the ACAVF and guides percutaneous therapy. Right heart catheterization (RHC) was performed in 19 cases and demonstrated elevated filling pressures in nine patients and normal pressures in 10 (Table 1).

Coronary angiography can identify the origin of a coronary fistula and assess hemodynamics, but may fail to demonstrate the relation to other structures and the drainage site [32]. Cardiac MRI and CT angiography have emerged as valuable modalities to demonstrate the size, course, anatomic connection, and other anatomic features of larger and more complex congenital and acquired coronary fistulas [32, 33] and provide valuable information necessary for surgical treatment [34–38]. None of the more recently reported ACAVFs due to inadvertent grafting of a coronary vein were evaluated by MRI or CT, most likely because these fistulas are smaller and better defined and because surgical closure has been replaced by percutaneous treatments.

Medical management and observation remain the treatment of choice for asymptomatic patients. Deligonul et al. [24] presented 2 cases of asymptomatic iatrogenic ACAVF, which closed spontaneously while the patients received medical management, supporting a more conservative approach to these patients. For symptomatic patients and those who are refractory to medical therapy, surgical closure of the ACAVF and grafting of the native artery have historically been preferred. With improvements in percutaneous methods, coil or balloon embolization, sometimes combined with stenting of the ungrafted artery, have become the new standard [18, 29]. In nearly half of the reported patients who were treated percutaneously (7 out of 15), the unbypassed vessel was not treated and the patient was treated with optimal medical therapy for coronary artery disease. These patients remained asymptomatic, suggesting that the shunt, rather than the unbypassed artery, might have been the underlying cause of the patients' symptoms.

Another advance in the treatment of this complication is the percutaneous approach to the ACAVF via the coronary sinus. Sheiban et al. [20] described a case in which a covered stent was deployed in the cardiac vein using this approach for the closure of an end-to-side anastomosis of the graft to the vein. Additionally, Lopez et al. [22] also used a coronary sinus approach to treat a side-to-side anastomosis of the proximal segment of a sequential graft that was anastomosed to a cardiac vein, with preservation of the distal end-to-side anastomosis to the coronary artery. 

## 4. Conclusion

Iatrogenic aorto-coronary arteriovenous fistula due to inadvertent anastomosis of a bypass graft to a cardiac vein is a rare complication that is probably more common than previously believed and may be a more frequent cause of graft failure and recurrent angina following CABG. Anatomical distortion of the surface of the myocardium, the presence of epicardial fat, and an intramyocardial course of the intended artery for grafting are predisposing factors.

Left heart catheterization is the diagnostic test of choice for this complication. Cardiac MRI and CTA have a more limited diagnostic role due to the smaller size of the fistula and its more easily defined course when compared to congenital and other types of acquired coronary artery fistulas. Asymptomatic patients should be observed and managed medically as they may have spontaneous closure of their fistulas. Percutaneous embolization with either detachable balloons or coils combined with stenting of the ungrafted artery is an effective and safe method of treatment for symptomatic patients.

## Figures and Tables

**Table 1:** Reports of inadvertent attachment of a bypass graft to a cardiac vein.

Author	Patient	Symptoms/onset after CABG	Murmur	Graft/Intended Artery/Actual Anastamosis	Shunt	Hemodynamics	CABG	Previous MI	Treatment
Vieweg [1]	53 M	CHF/6 weeks	Continuous 2nd LICS	SVG/LAD/anterior cardiac vein	Shunt by hydrogen inhalation; normal pulse ox	Mild elevation of right heart pressures	First	No	Graft removal; regrafting of SVG to LAD
Lawrie et al. [2]	44 M	Angina pectoris; <3 months	Systolic; base to neck	SVG/LAD/LAD vein	Not mentioned	Not mentioned	Third	No	Graft ligation; SVG to LAD
Treistman et al. [3]	55 M	SVT, palpitations, syncope; 3.5 years	Continuous 3rd LICS	SVG/LAD/anterior interventricular vein	Normal oximetry	Mild elevation of right heart pressures	First	Anterolateral MI	None
Klinke et al. [4]	40 M	Angina pectoris; 5 months	Not mentioned	SVG/LAD/anterior interventricular vein	Coronary sinus 02 saturation 90%	Normal	First	No	CABG
Starling et al. [5]	47 M	None reported. Anterior ischemia; 3 weeks	Continuous 2nd LICS to apex	SVG/LAD/anterior cardiac vein	None	Normal	First	Posterolateral MI	Fistula ligation; SVG to diagonal
Starling et al. [5]	66 M	Asymptomatic	Continuous 2nd and 3rd LICS to the apex	SVG/proximal LAD to distal LAD/anterior cardiac vein distal LAD	None	Normal	First	Anteroseptal MI	Observation
Grollman Jr. et al. [6]	52 M	Fatigue, dyspnea; 1 year	None	SVG/anterolateral branch of Cx/anterior interventricular vein	1.1 : 1	RVEDP 8 mmHg LVEDP 25 mmHg	First	Anteroapical MI	Percutaneous occlusion of SVG with 2 coils
Hubert et al. [7]	55 M	CHF, VT; 1 month	Continuous ULSB	SVG/LV branch of RCA PL of Cx/LV branch posterior interventricular vein	Normal oximetry	RA 20 mmHg	First	Inferior MI	Ligation of fistula
Przybojewski [8]	43 M	Angina pectoris; 10 days	Continuous 2nd and 3rd LICS	SVG/LAD/LAD vein	Not mentioned	Not mentioned	First	Anterolateral MI	Ligation; repeat CABG
Goldbaum et al. [9]	53 M	Angina pectoris, exertional dyspnea; 4 years	None	SVG/LAD/anterior interventricular vein	Small; not quantified	PA 42/19 LVEDP 19	First	Anterior MI	PTCA of LAD; percutaneous occlusion of SVG with coils
Ross and Jang [10]	44 M	Anginal pectoris; onset not mentioned	Systolic; ULSB	SVG/intermediate or Cx/left marginal vein	1.4 : 1	Normal	Second	Inferior MI	None reported
Jost et al. [11]	57 M	Angina pectoris; 2 years	Not mentioned	SVG/LAD/anterior cardiac vein	18% of pulmonary flow	Not mentioned	First	No	Embolization with silicone balloon
Graeb et al. [12]	56 F	Angina pectoris; 1 year	Not mentioned	SVG/PDA/PDV	Small	Normal	First	No	Balloon embolization of PDA (unsuccessful)
Marin-Neto et al. [13]	57 M	Dyspnea, chest pain; 1 month (no ischemia detected)	Systolic; pulmonic area	SVG/first diagonal/anterior cardiac vein	23% of pulmonary flow	Normal	First	Inferior MI	None
Marin-Neto et al. [13]	84 M	Angina pectoris; 14 months	Not mentioned	SVG/first diagonal/anterolateral coronary vein	12% of pulmonary flow	Not mentioned	First	No	PTCA of new RCA lesion, no treatment of fistula
Scholz et al. [14]	49 M	Angina pectoris 15 months	Systolic ULSB	SVG/OM1, OM2/OM1, coronary vein	Small	Normal	First	No	Observation
Calkins Jr. et al. [15]	51 F	Angina pectoris; 2-3 months	None	SVG/OM1, OM2/OM1 coronary vein	None	RV 55/15 PA 55/17	Second	No	Coil embolization
De Marchena et al. [16]	73 M	Diminished exercise capacity, dyspnea; 2 months	None	LIMA/LAD/great cardiac vein	Small	RV 68/12 RA 12 mmHg	First	No	Observation
Khunnawat et al. [17]	75 F	Dyspnea; 10 years	S3, no murmur	SVG/RCA/cardiac vein	Not mentioned	Not mentioned	First	LBBB on EKG	Not mentioned
Khunnawat et al. [17]	57 M	Dyspnea; 6 years	None	LIMA/LAD/LAD cardiac vein	Not mentioned	Not mentioned	First	Not mentioned	Not mentioned
Maier et al. [18]	50 M	Dyspnea and Angina pectoris; 2 years	Systolic murmur at ULSB	SVG/D1/coronary vein	Large left to right	Pulmonary C.O. 6.6 L/mm, systemic CO 4.8 L/mm, normal pulmonary pressures	First	Not mentioned	PTCA; failed percutaneous coil embolization led to percutaneous transcatheter detachable balloon of SVG to D1
Patterson et al. [19]	67 M	angina Pectoris; 7 months	Not mentioned	LIMA-RIMA/PL/PL vein	Present	Not mentioned	First	Not mentioned	PCI to revascularize Cx; then coil embolization of RIMA
Sheiban et al. [20]	73 M	Positive stress test and angina with exertion; 2 months	Not mentioned	LIMA/LAD/GCV	Present “arteriovenous steal” Moderate L-R Shunt	Not mentioned	First	No	PCI with DES of LAD and PCI with covered stent of GCV through coronary sinus
Hmem et al. [21]	76 M	Dyspnea, LE edema, CHF/RHF; 2 months	None	LIMA/LAD/LIMA/cardiac vein	Not mentioned	Not mentioned	First	AS Q waves	Coil embolization to proximal LIMA
Lopez et al. [22]	74 M	Rest angina; 3 months	Not mentioned	SVG-OM2-OM3/SVG-L marginal vein of OM3	No significant left to right shunt	Not mentioned	Second	Not mentioned	PCI, embolization of marginal vein
Braun et al. [23]	58 M	Angina; 6 months	Not mentioned	LIMA/LAD/LIMA/cardiac vein	Not mentioned	Not mentioned	Second	Not mentioned	RCA-PTCA; coil embolization
Deligonul et al. [24]	66 M	Asymptomatic	None	LIMA/LAD/anterior interventricular vein	Small	None	First	No	Spontaneous closure
Deligonul et al. [24]	57 M	Asymptomatic	None	LIMA/LAD/anterior interventricular vein	Small	None	First	No	Spontaneous closure
Miranda et al. [25]	66 M	Angina; 2 weeks	Not mentioned	LIMA/LAD/anterior interventricular vein	Small	Pulmonary artery pulse ox 66%	Second	No	PTCA to diagonal branch graft followed by balloon occlusion of fistula
Peregrin et al. [26]	54 M	Unstable angina; 1 year	Systolic/diastolic left parasternal	Graft/diagonal branch/graft vena cordis magna	Not mentioned	Not mentioned	First	Inferior MI	PTCA of RCA and balloon occlusion of AV fistula
Cardoso [27]	55 F	Angina	Continuous second left intercostals space	SVG/LAD/LAD vein	Aortic oxygen saturation 92%, coronary sinus saturation 80%, SVC saturation 73%	LV 171/19, RV 43/12, mean PCWP 21	First	No	Not mentioned
White et al. [28]	56 M	Angina and troponin elevation; 5 years	Not mentioned	LCX-vein graft-marginal/LCX-coronary sinus	None	None	First	No	MRI; coil embolization
Thomas et al. [29]	76 M	Angina with exertion; 3 months	Not mentioned	LIMA/LAD/AIV	Not mentioned	Not mentioned	First	Not mentioned	PCI with stenting and PCI with embolization
Mukhopadhyay et al. [30]	60 M	Exertional angina	Not mentioned	LIMA/LAD/cardiac vein	Left to right shunt	RH and PA pressures normal, 02 sat was 84% on step up in coronary sinus and 72% in the RA to 80% in PA	First	Not mentioned	PCI with failed coil embolization, deferred to surgical correction
Jung et al. [31]	50 M	Asymptomatic	Not mentioned	LIMA/LAD/GCV	Not mentioned	ECHO showed LV hypokinesis and LV systolic dysfunction.	First	Not mentioned	CT angiogram; PCI/DES and Coil embolization
**Current Case**	69 M	Angina with exertion left arm radiation; 3 months	None	LIMA/LAD/LIMA/cardiac vein	None	Aorta 119/54, mean 77, LV systolic: 130, LVEDP: 14, LV-angiography EF = 55%	First	No	PCI/DES and coil embolization to LIMA
